# Genomic Epidemiology of Azithromycin-Nonsusceptible *Neisseria gonorrhoeae*, Argentina, 2005–2019

**DOI:** 10.3201/eid2709.204843

**Published:** 2021-09

**Authors:** Ricardo Ariel Gianecini, Tomas Poklepovich, Daniel Golparian, Noelia Cuenca, Ezequiel Tuduri, Magnus Unemo, Josefina Campos, Patricia Galarza, Gonococcal Antimicrobial Susceptibility Surveillance Programme—Argentina

**Affiliations:** Instituto Nacional de Enfermedades Infecciosas—Administración Nacional de Laboratorios e Institutos de Salud Dr. Carlos G. Malbrán, Buenos Aires, Argentina (R.A. Gianecini, T. Poklepovich, N. Cuenca, E. Tuduri, J. Campos, P. Galarza);; World Health Organization Collaborating Centre for Gonorrhoea and Other STIs, Örebro University, Örebro, Sweden (D. Golparian, M. Unemo)

**Keywords:** whole-genome sequencing, gonorrhea, azithromycin, antimicrobial resistance, treatment, AMR, sexually transmitted infections, *Neisseria gonorrhoeae*, bacteria, bacterial infections, Argentina, genomics

## Abstract

Azithromycin-nonsusceptible *Neisseria gonorrhoeae* strains are an emerging global public health threat. During 2015–2018, the prevalence of azithromycin-nonsusceptible gonococcal infection increased significantly in Argentina. To investigate the genomic epidemiology and resistance mechanisms of these strains, we sequenced 96 nonsusceptible isolates collected in Argentina during 2005–2019. Phylogenomic analysis revealed 2 main clades, which were characterized by a limited geographic distribution, circulating during January 2015–November 2019. These clades included the internationally spreading multilocus sequence types (STs) 1580 and 9363. The ST1580 isolates, which had MICs of 2–4 μg/mL, had mutations in the 23S rRNA. The ST9363 isolates, which had MICs of 2–4 or >256 μg/mL, had mutations in the 23S rRNA, a mosaic *mtr* locus, or both. Identifying the geographic dissemination and characteristics of these predominant clones will guide public health policies to control the spread of azithromycin-nonsusceptible *N. gonorrhoeae* in Argentina.

Gonorrhea, caused by infection with the bacterium *Neisseria gonorrhoeae*, is the second most prevalent bacterial sexually transmitted infection (STI) globally (*1*,*2*). The World Health Organization (WHO) estimated that in 2016, a total of 86.9 million incident gonorrhea cases occurred among persons 15–49 years of age, including 13.8 million cases in the WHO Region of the Americas (*1*). Researchers have documented antimicrobial resistance (AMR) to all drugs used to treat gonorrhea (*2*,*3*). Ceftriaxone, an extended-spectrum cephalosporin, is the last option for first-line empirical treatment, but the emergence of ceftriaxone resistance has raised concerns about future treatments (*2*,*4*). Consequently, WHO guidelines and national guidelines of many countries now recommend a combination of ceftriaxone (250 mg–1 g) and azithromycin (1–2 g) as first-line treatment for uncomplicated gonorrhea (*5*,*6*). However, in 2016 Fifer et al. (*7*) reported the failure of dual therapy. Two years later, a gonococcal strain with ceftriaxone resistance and high-level azithromycin resistance was isolated in Australia and England (*8*–*10*). In recent years, international reports of azithromycin-resistant *N. gonorrhoeae* have substantially increased (*2*,*3*,*11*,*12*). The WHO Global Gonococcal Antimicrobial Surveillance Program showed that in 2016, a total of 48.4% of reporting countries had an >5% increase in rates of azithromycin resistance (*3*). 

Argentina has reported low azithromycin resistance levels since the early 2000s (*13*). In Argentina, the proportion of azithromycin-nonsusceptible isolates (i.e., requiring MICs ˃1 μg/mL) increased from 0.1% in 2015 to 4.3% in 2018 (p˂0.01) (*14*). The Clinical and Laboratory Standards Institute currently states a susceptible-only breakpoint for azithromycin (*15*); for simplicity, we refer to these isolates as resistant. High-level azithromycin-resistant isolates requiring MICs >256 μg/mL have emerged in several countries, including Argentina (*16*–*20*). Azithromycin resistance threatens the effectiveness of dual antimicrobial gonorrhea treatment.

Whole-genome sequencing (WGS) provides higher resolution and accuracy than other typing methods, making it an ideal method to study the dissemination and transmission dynamics of *N. gonorrhoeae* strains on a national and international level (*21*,*22*). Furthermore, WGS data offer insights into AMR determinants, thereby enabling prediction, enhanced detection, and characterization of high-risk clones (*22*,*23*). Several studies have found *N. gonorrhoeae* lineages and clones driving AMR transmission among *N. gonorrhoeae* strains within local, national, and international networks (*16*,*17*,*24*–*26*). Genomic surveillance provides information on current and emerging trends of circulating strains. Phenotypic, epidemiologic, and genomic surveillance data are critical for designing public health interventions and treatment strategies. Genomic approaches, including molecular epidemiology and detection of AMR determinants, are crucial for monitoring resistance to first-line drugs. We examined the genomic background of azithromycin-resistant *N. gonorrhoeae* isolates with MICs >2 μg/mL collected throughout Argentina during 2005–2019.

## Materials and Methods

We examined 96 azithromycin-resistant *N. gonorrhoeae* isolates (MICs >2 μg/mL) from male and female patients treated at STI hospitals throughout Argentina. We selected 95 isolates from 8,002 consecutive isolates collected through the Gonococcal Antimicrobial Susceptibility Surveillance Programme—Argentina during January 2005–November 2019; we also included an isolate with high-level azithromycin resistance cultured in 2001 (*20*). We confirmed the *N. gonorrhoeae* species by culture on selective agar media, microscopic analysis using Gram staining, rapid oxidase positivity, superoxol test, carbohydrate utilization test, and matrix-assisted laser desorption/ionization time-of-flight mass spectrometry (microflex LT/SH; Bruker Daltonik, https://www.bruker.com) (*27*). The study was approved by the Research Ethics Committee of the Hospital General de Agudos “Bernardino Rivadavia” (Buenos Aires, Argentina). MIC determinations and whole-genome sequencing for all isolates were conducted using methods previously described (Appendix). 

### WGS Analysis

We identified AMR determinants (i.e., the *mtrR*-35A, *mtr*_120_, and mosaic *N. meningitidis–*like *mtrR* mutations) in addition to the MtrR A39T and G45D amino acid mutations in silico from WGS data, as described (*26*,*28*). We aligned and compared the *mtr* locus and *rplD*, *rplV*, and *macAB* sequences with the *N. gonorrhoeae* FA1090 reference genome (GenBank accession no. AE004969). To identify the frequency of 23S rRNA A2059G and C2611T mutations (named using *Escherichia coli* numbering), we mapped sequence reads against a single copy of the FA1090 23S rRNA gene using Burrow-Wheeler Aligner version 0.7.17 (http://bio-bwa.sourceforge.net) with the default settings. We determined base counts using a custom script, enabling the estimation of the proportion of copies with the A2059G, C2611T, or both mutations. We examined additional macrolide resistance genes (e.g., *ereA*, *ereB*, *ermA*, *ermB*, *mefA*, *mefB*, *msrA*, and *msrC*) using ARIBA version 2.14.4 and the ResFinder (https://cge.cbs.dtu.dk/services/ResFinder) and CARD (https://card.mcmaster.ca) databases (*29*). We identified alleles in silico from WGS data using *N. gonorrhoeae* multiantigen sequence typing (NG-MAST), multilocus sequence typing (MLST), and N. gonorrhoeae sequence typing for antimicrobial resistance (NG-STAR). We used the MLST (https://pubmlst.org/neisseria), NG-MAST (http://www.ng-mast.net), and NG-STAR (https://ngstar.canada.ca) databases to assign allele numbers and sequence types (ST)s (*30*,*31*). We grouped closely related NG-MAST STs using a previously described genogroup definition (*28*).

For phylogenetic analysis, we identified single-nucleotide polymorphisms (SNPs) in sequence reads mapped against the WHO P reference genome using the variant calling tool Snippy version 4.4.5 (https://github.com/tseemann/snippy). We identified and filtered recombinant regions using Gubbins version 2.1.0 (Sanger, https://sanger-pathogens.github.io/gubbins); the resulting core SNP alignment consisted of 9,415 sites. We used IQ-tree version 1.6.1 (http://www.iqtree.org) to infer a maximum-likelihood tree from the whole-genome SNP alignment with a generalized time-reversible model of evolution using gamma correction for among-site rate variation with 4 rate categories; branch support was estimated by bootstrap analysis of 10,000 replicates (*32*). We visualized the resulting phylogeny with Figtree version 1.4.4 (http://tree.bio.ed.ac.uk/software/figtree) and phandango (*33*). We clustered sequences using RAMI with a branch length threshold of 0.01 (*34*). For comparison, we selected international isolates and publicly available genomic data on the basis of MICs, MLST STs (i.e., ST9363 and ST1580), and NG-MAST genogroups (i.e., G470 and G12302) from the National Center for Biotechnology Information (https://www.ncbi.nlm.nih.gov), European Molecular Biology Laboratory (https://www.embl.org), and the DNA Data Bank of Japan (https://www.ddbj.nig.ac.jp). We found 17 genomes from the United Kingdom, 3 from Canada, 3 from Scotland, 17 from Australia, 28 from the United States, 7 from Brazil, and 11 from Norway (*16*,*17*,*24*,*25*,*35*–*37*). We generated a phylogenetic tree of 86 international and 96 isolates from Argentina as described for domestic isolates and visualized the tree in Figtree version 1.4.4. Sequence reads are available from the European Nucleotide Archive (accession no. PRJEB41007).

## Results

### Patient Data

The 96 *N. gonorrhoeae* isolates were collected from male (90.6%) and female (6.3%) patients; sex was unreported for 3.1% of patients. Patient age was reported for 88 (91.7%) isolates. Patients were 4–47 years of age (mean 24.3 years of age); 79.5% were <30 years of age. In total, 72 isolates were cultured from the urethra, 11 from urine, 3 from the cervix, 2 from the vagina (in children 4 and 6 years of age), 1 from the pharynx, and 7 from an unreported site.

**Figure 1:**
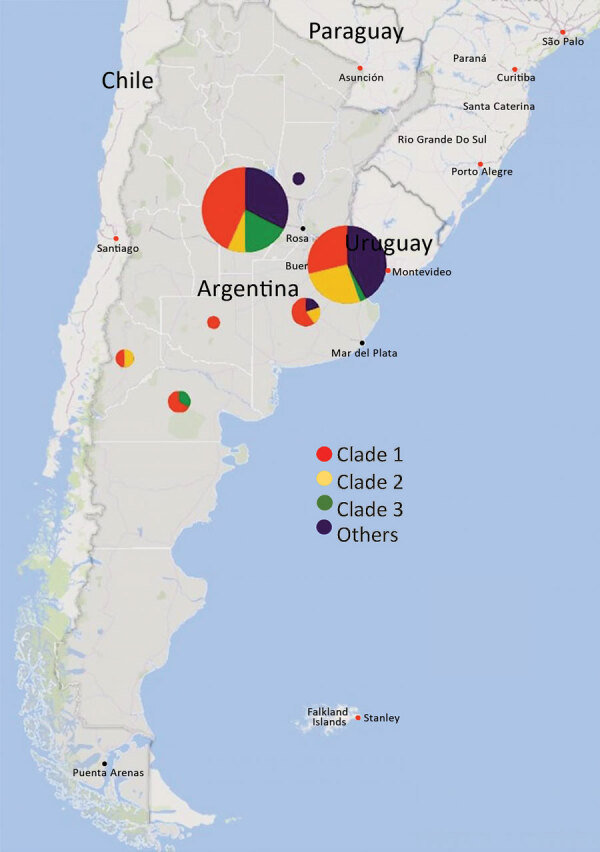
Geographic distribution of *Neisseria gonorrhoeae* isolates with azithromycin MICs of >2 μg/mL, Argentina, January 2005–November 2019. Circle size corresponds to the number of isolates in each location. Circle colors indicate the proportion of isolates belonging to the 3 main genomic clades compared with other clades.

The isolates were collected in 7/24 provinces. Among these, Córdoba and Ciudad Autónoma de Buenos Aires (CABA), 2 of the most populated provinces in Argentina, had the highest percentage of isolates (Córdoba had 47.9%; CABA had 39.6%) (Figure 1). We observed a lower percentage of isolates from the provinces of Buenos Aires (5.2%), Rio Negro (3.1%), Neuquén (2.1%), La Pampa (1.0%), and Santa Fe (1.0%).

### Antimicrobial Susceptibility of *N. gonorrhoeae* Isolates

Overall, 78 (81.3%) isolates had azithromycin MICs of 2–4 μg/mL, 13 (13.5%) had MICs of 8–16 μg/mL, and 5 (5.2%) had MICs of >256 μg/mL (Table 1). Among 5 isolates with MICs >256 μg/mL, 3 were collected in CABA in 2001 (n = 1) and 2019 (n = 2); the other 2 isolates were collected in Buenos Aires in 2018 and Córdoba in 2019. All 96 azithromycin-resistant isolates were susceptible to ceftriaxone, cefixime, and spectinomycin. However, 2 isolates collected in 2016 from Córdoba, each had a MIC of 4 μg/mL, showed decreased susceptibility to ceftriaxone (MIC = 0.06 μg/mL) and cefixime (MIC = 0.125 μg/mL) (Table 2).

**Table 1:** Characteristics of 96 azithromycin-resistant *Neisseria gonorrhoeae* isolates, Argentina, January 2005–November 2019*

Characteristics	MICs for azithromycin, μg/mL		
	2–4	8–16	>256
Total	78	13	5
Province	Buenos Aires, CABA, Córdoba, Neuquén, La Pampa, Río Negro, Santa Fe	CABA, Córdoba	Buenos Aires, CABA, Córdoba
Resistance determinants			
23S rRNA (no. mutated alleles; total no. isolates)	C2611T (4; 58); C2611T (1; 1)	C2611T (4; 12); C2611T (3; 1)	A2059G (4; 5)
MtrR protein (no. isolates)	A-deletion (12)†; *N. meningitidis*–like (14); G45D (41);* mtr_120 _*(0)‡	A-deletion (6); G45D (3);* mtr_120_* (0)	*N. meningitidis*–like (4); G45D (1);* mtr_120_* (0)
Mosaic *mtr* locus (no. isolates)	*mtrC* (14); *mtrD* (14); *mtrE* (13)	*mtrC* (0); *mtrD* (0); *mtrE* (0)	*mtrC* (4); *mtrD* (4); *mtrE* (4)
ST			
*N. gonorrhoeae *multiantigen sequence typing (no. isolates)	ST470 (23); ST20102 (7); ST696 (4); ST12302 (4); ST11062 (3); other STs (37)	ST18761 (3); ST20104 (3); singleton STs (7)	ST3935 (2); ST20106 (2); ST696 (1)
Multilocus sequence typing (no. isolates)	ST1580 (39); ST1584 (10); ST9363 (10); ST1901 (8); other STs (11)	ST1901 (6); ST1580 (3); ST13844 (3); ST13594 (1)	ST9363 (4); ST1580 (1)
*N. gonorrhoeae* sequence typing for antimicrobial resistance (no. isolates)	ST1038 (30); ST179 (10); ST168 (5); ST3200 (4); other STs (29)	ST27 (4); ST2728 (3); ST1038 (2); singleton STs (4)	ST1993 (2); ST2906 (1); ST3194 (1); ST3199 (1)

*CABA, Ciudad Autónoma de Buenos Aires; ST, sequence type. †Deletion of A in 13-bp inverted repeat sequence of the *mtrR* gene. ‡C-to-T transition mutation 120 bp upstream of the *mtrC* start codon.

**Table 2:** Antimicrobial susceptibility of 96 azithromycin-resistant *Neisseria gonorrhoeae* isolates, Argentina, January 2005–November 2019*

Antimicrobial drug	Azithromycin MICs, μg/mL (no. isolates)						
	2–16 (91)					>256 (5)	
	MIC_50_	MIC_90_	Range	Resistance, %		MIC	Resistance, %
Ciprofloxacin	0.004	16	0.001–32	28.6		0.002–4	20
Tetracycline	1	2	0.125–4	25.3		0.5–2	20
Benzylpenicillin	1	2	0.25–8	14.3		0.5–1	0
Ceftriaxone	0.004	0.03	0.002–0.06	0		0.004–0.016	0
Cefixime	0.016	0.03	0.004–0.125	0		0.008–0.03	0
Spectinomycin	32	32	16–32	0		32	0
Gentamicin	8	8	4–16	0		8–16	0

*MIC_50_, MIC for 50% of isolates; MIC_90_, MIC for 90% of isolates.

### Molecular AMR Determinants

All 5 isolates with MICs of >256 μg/mL had the A2059G mutation in all 4 23S rRNA alleles, whereas none of the 91 isolates with MICs of 2–16 μg/mL had this SNP (Table 1). Most (72; 75%) isolates with MICs of 2–16 μg/mL had the 23S rRNA C2611T mutation. Nearly all (70; 97.2%) of these isolates had the C2611T mutation in all 4 23S rRNA alleles, except for 2 isolates: 1 with a single mutated allele that had a MIC of 4 μg/mL and 1 with 3 mutated alleles that had a MIC of 8 μg/mL. Interspecies mosaics in the *mtr* locus (which encodes the tripartite MtrCDE efflux pump), as well as mutations in the *mtrR* promoter, coding region, or both, have been associated with increased azithromycin MICs (*38*–*40*). Among the 80 (83.3%) isolates with *mtrR* mutations, 17 (17.7%) had an *mtrR*-35A promoter deletion, 44 (45.8%) had an MtrR G45D amino acid mutation, 1 (1.0%) had an *mtrR*-35A deletion and MtrR G45D substitution, and 18 (18.8%) had a mosaic *N. meningitidis*–like *mtrR* promoter. We did not identify any isolates with the *mtr*_120_ mutation. Eighteen isolates, all of which had MICs of 2–4 μg/mL, had no 23S rRNA mutations; however, 13 contained a mosaic *mtrR* promoter and 5 had a *mtrR*-35A deletion. Among 18 isolates with mosaic *mtrR* promoters, 100% also had mosaic sequences in the *mtrD*, 100% in *mtrC*, and 94.4% in *mtrE* loci. Fifteen isolates with a mosaic *mtrD* allele had sequences identical to the *N. meningitidis*–like mosaic previously described (*39*,*40*); 2 isolates had sequences sharing 97.8% identity and 1 had a sequence sharing 97.3% identity with the *N. meningitidis*–like mosaic (Appendix Figure 1). Isolates containing a mosaic-like *mtr* locus had MICs of >2 to >256 μg/mL. Isolates with MICs of >256 μg/mL also contained the 23S rRNA A2059G mutation.

We did not find any mutations associated with macrolide resistance in the *rplD* gene, which encodes ribosomal protein L4, or the *rplV* gene, which encodes ribosomal protein L22 (*23*). In addition, we did not find AMR mutations in *macAB*, which encodes the MacA-MacB efflux pump, or the acquired macrolide resistance genes, *ere*, *mef*, *erm*, *mph*, and *msr* (*38*). An isolate that had a MIC of 2 μg/mL had an unclear resistance mechanism.

### Molecular Epidemiology and Phylogenomic Analysis

Among the 96 *N. gonorrhoeae* isolates, we observed 42 NG-MAST STs, including 21 new STs and 25 STs represented by single isolates. We found 24 isolates belonging to ST470, 7 belonging to ST20102, 6 belonging to ST696, 4 belonging to ST12302, and 4 belonging to ST20104. We found 3 NG-MAST genogroups comprising >3 isolates: 33 belonged to G470, 10 belonged to G12302, and 10 belonged to G20102. We also documented 14 MLST STs, including 2 new STs and 8 STs represented by single isolates. We found 43 isolates belonging to ST1580, 14 belonging to ST1901, 14 belonging to ST9363, and 10 belonging to ST1584. NG-STAR showed 32 types, of which 11 were new and 20 were represented by single isolates. We found 32 isolates belonging to NG-STAR type 1038, 10 belonging to type 179, 5 belonging to type 168, and 5 belonging to type 3200.

**Figure 2:**
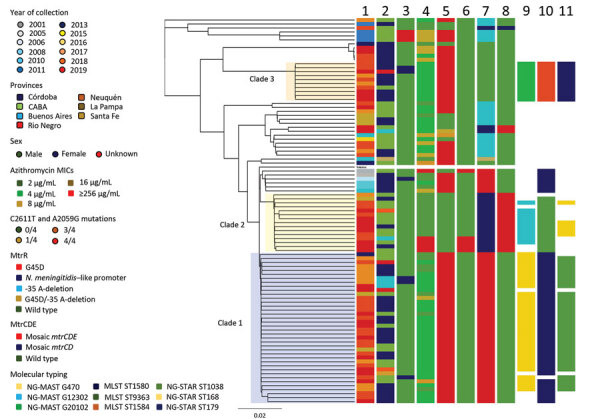
Phylogenomic tree of 96 *Neisseria gonorrhoeae* isolates with azithromycin MICs of >2 μg/mL, Argentina, January 2005–November 2019. Lane 1, year; lane 2, province; lane 3, sex; lane 4, azithromycin MICs; lane 5, 23S C2611T; lane 6, 23S A25059G; lane 7, MtrR; lane 8, MtrCDE; lane 9, NG-MAST; lane 10, MLST; lane 11, NG-STAR. Scale bar indicates substitutions per site. CABA, Ciudad Autónoma de Buenos Aires; MLST, multilocus sequence typing; NG-MAST, *N. gonorrhoeae* multiantigen sequence typing; NG-STAR, *N. gonorrhoeae* sequence typing for antimicrobial resistance; ST, sequence type.

Analysis of the phylogenomic tree revealed 14 clades. In total, 63 (65.6%) isolates were grouped into 3 clades, each containing 10–38 isolates (Figure 2) (https://microreact.org/project/AZM_Project/006b822d). The remaining 33 isolates were singletons or belonged to smaller clonal groups of 2–6 isolates each. 

Clade 1 comprised 38 isolates, most of which belonged to NG-MAST G470 (86.8%), MLST ST1580 (97.4%), or NG-STAR ST1038 (84.2%). Clade 1 isolates had mean SNP difference of 8.5 (range 0–39). The isolates required MICs of 2–16 μg/mL; most (76.3%; 29/38) required an MIC of 4 μg/mL. The oldest isolate in clade 1 was identified in CABA in 2013. The proportion of clade 1 isolates increased significantly from 1.0% (1/96) in 2013 to 11.4% (11/96) in 2019 (p<0.05). Clade 1 was dominated by isolates from Córdoba (52.6%; 20/38) and CABA (28.9%; 11/38) but also included isolates obtained in 4 additional provinces. In total, 92.1% of the clade 1 isolates were from male patients and 7.9% were from female patients. Clade 1 isolates were characterized by the 23S rRNA C2611T mutation in all 4 alleles and the MtrR G45D amino acid mutation. 

Clade 2 comprised 15 isolates that mainly belonged to NG-MAST G12302 (66.7%) and MLST ST9363 (93.3%). Clade 2 isolates had a mean SNP difference of 13.1 (range 0–33). All clade 2 isolates were cultured from men. Most (73.3%; 11/15) required an MIC of 2 μg/mL, and 26.7% (4/15) required MICs of >256 μg/mL. The first clade 2 isolate was detected in Córdoba in 2016; during 2017–2019, isolates were mainly detected in CABA (71.4%; 10/14), except for 2 isolates detected in Córdoba, 1 in Neuquén, and 1 in Buenos Aires. Clade 2 isolates did not have the 23S rRNA C2611T mutation but possessed the mosaic *mtrR* promoter and *mtrCDE* locus. In addition, isolates requiring MICs of >256 μg/mL had the 23S rRNA A2059G mutation in all 4 alleles. 

Clade 3 was composed of 10 isolates belonging to NG-MAST G20102 and MLST ST1584. Clade 3 isolates had a mean SNP difference of 1.1 (range 0–2). Eight isolates were collected in Córdoba, 1 in CABA, and 1 in Río Negro during 2017–2019; of these, 8 were from men. All isolates required an MIC of 4 μg/mL and possessed the 23S rRNA C2611T mutation in all 4 alleles.

**Figure 3:**
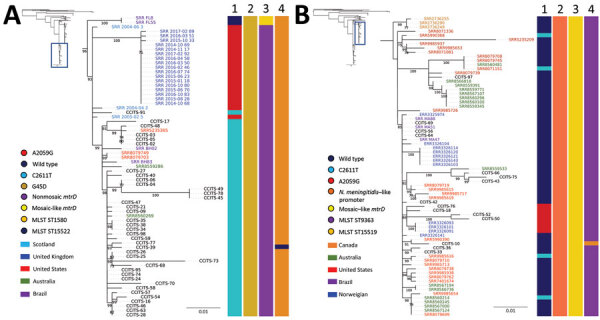
Phylogenomic tree of *Neisseria gonorrhoeae* isolates with azithromycin MICs of >2 μg/mL, 2004–2017. A) MLST ST1580 and NG-MAST genogroup 470 isolates from Argentina in the context of selected isolates from Scotland (2004–2005), the United States (2016), Australia (2017), Brazil (2015–2016), and the United Kingdom (2014–2017). B) MLST ST9363 and NG-MAST genogroup G12302 isolates from Argentina in the context of selected isolates from Australia (2017), the United States (2014–2017), Brazil (2015–2016), Norway (2017), and Canada (2013–2014). Lane 1, 23S rRNA; lane 2, *mtrR*; lane 3, *mtrD*; lane 4, MLST. Labels indicate isolate identity; font colors indicate country of isolation. Bar colors indicate distribution of mutations. Insets indicate relationship of sequences to larger phylogenetic tree. Scale bar indicates substitutions per site. MLST, multilocus sequence typing; NG-MAST, *N. gonorrhoeae* multiantigen sequence typing.

To investigate the international context of the 2 major MLST STs in Argentina, including azithromycin-resistant ST1580 and ST9363, we conducted a phylogenomic analysis using SNPs (Figure 3) (https://microreact.org/project/AZM_Project_2/7a2032e2). The ST1580 isolates from Argentina clustered with isolates from the United States, the United Kingdom (particularly Scotland), Australia, and Brazil. The mean pairwise SNP differences between ST1580 isolates from Argentina and other countries were 6.8 (range 1–23) for the isolates from the United States, 6.9 (range 1–22) for isolates from Australia, 7.9 (range 4–22) for isolates from Scotland, 11.4 (range 4–28) for isolates from Brazil, and 16.8 (range 13–31) for isolates from the United Kingdom (excluding Scotland). Isolates from Scotland and the United Kingdom had MICs of >256 μg/mL whereas isolates from the United States, Australia, and Brazil had MICs of 2–8 μg/mL. All isolates with MICs of 2–8 μg/mL had the 23S rRNA C2611T mutation and all isolates with MICs of >256 μg/mL had the A2059G mutation. In addition, 2 isolates from Brazil had mosaic *mtrD* alleles, but no mutations in the 23S rRNA gene; these isolates had MICs of 2 μg/mL. The ST9363 isolates from Argentina clustered with other ST9363 isolates from the United States, Australia, Canada, Brazil, and Norway. ST9363 isolates from Argentina had a mean pairwise SNP difference of 7.7 (range 0–20) with isolates from Brazil, 10.1 (range 1–23) with isolates from Norway, 12.5 (range 5–25) with isolates from Canada, 13.1 ( range 2–42) with isolates from the United States, and 14.8 (range 2–35) with isolates from Australia. All isolates had mosaic *mtrR* promoters and *mtrD* alleles. All isolates with MICs of >256 μg/mL had the 23S rRNA A2059G mutation and 4 isolates with MICs of 8–16 μg/mL had the 23S rRNA C2611T mutation.

## Conclusion

We characterized the genomes of azithromycin-resistant *N. gonorrhoeae* isolates collected in Argentina during 2005–2019. Phylogenomic analysis showed that isolates from Argentina clustered into distinct clades, including 3 clades comprising 63 (65.6%) isolates collected during 2016–2019. All isolates also were resistant to benzylpenicillin, tetracycline, and ciprofloxacin, or some combination, but susceptible to ceftriaxone and cefixime.

In Argentina, dual therapy is recommended as first-line treatment for uncomplicated gonorrhea, according to the WHO guidelines (*6*). The Argentine Ministry of Health and the Sociedad Argentina de Infectología recommend a single 1-g dose of azithromycin monotherapy for the treatment of *Chlamydia trachomatis* and *Mycoplasma genitalium* infections (*41*,*42*). These guidelines also recommend antimicrobial treatment for suspected infections. Azithromycin has a long half-life, resulting in detectable drug concentrations in human plasma for up to 14 days (*43*). Undiagnosed *N. gonorrhoeae* infections concurrent with the treatment of *C. trachomatis* and *M. genitalium* infections might lead to prolonged exposure to subinhibitory concentrations of azithromycin, thereby prompting the induction of or selection for resistance genes. In the United States and United Kingdom, dual therapy is no longer the first-line treatment. Instead, high-dose ceftriaxone monotherapy (500 mg in the United States or 1 g in the United Kingdom) is now recommended for treatment of uncomplicated gonorrhea (*44*,*45*). Moreover, additional treatment with doxycycline (100 mg 2×/d for 7 d) is recommended if chlamydial infection has not been excluded (*44*). Similar empirical antimicrobial therapies for gonorrhea and chlamydial infections might be of benefit in Argentina to reduce patient exposure to azithromycin and avoid the emergence of resistant gonococcal strains.

Azithromycin resistance (i.e., MICs of >2 μg/mL) in *N. gonorrhoeae* has been mainly associated with mutations in the 23S rRNA target (*38*). The 23S rRNA A2059G mutation causes high-level resistance (i.e., MICs of >256 μg/mL) and the C2611T mutation causes low-level resistance (i.e., MICs of 2–16 μg/mL) (*38*). We found that 75% of isolates had the C2611T mutation. These isolates were phylogenetically diverse; however, clade 1, which was predominated by MLST ST1580 and NG-MAST G470, comprised 38 (52.8%) isolates. NG-MAST ST470 has been associated with high-level resistance to azithromycin in Scotland (*18*). In addition, NG-MAST ST470 has >99% similarity to ST9768, which caused an outbreak of high-level azithromycin-resistant *N. gonorrhoeae* in the United Kingdom (*16*). Previous gonococcal evolution studies have estimated that ≈4 (range 0–14) SNPs occur per year per genome, enabling phylogenetic analysis (*21*). Isolates from Argentina differed from isolates from Scotland by >4 (mean 7.9) SNPs and the United Kingdom by 13 (mean 16.8) SNPs. In addition, NG-MAST ST470 isolates from the United States, Brazil, and Australia, all of which showed low-level resistance to azithromycin, were closely related to isolates from Argentina (mean 7–11 SNPs). These findings support the hypothesis that NG-MAST G470 strains from Argentina might be descended from 1 lineage of the ST470 clone, which has spread internationally and can develop high-level and low-level resistance to azithromycin. Previous research, especially that of Unemo et al. (*38*), hypothesized that gonococcal antimicrobial-resistant strains emerge through genetic events, such as horizontal gene transfer or spontaneous mutations; these strains can spread quickly within a geographic region through sexual networks. Furthermore, compensatory mutations or gene exchange might have preserved this lineage in Argentina. The presence of additional STs, such as the co-circulation of MLST ST1584 and MLST ST1580 (NG-MAST G470), suggests that novel introductions also have occurred.

The *mtr* locus recently has been described as a hotspot for genetic recombination; mosaic-like *mtr* loci are associated with decreased susceptibility to azithromycin (i.e., MICs of 1–4 μg/mL) and contribute to the survival and transmission of *N. gonorrhoeae* (*39*,*40*, *46*). Most clade 2 isolates were associated with MLST ST9363 and had a mosaic-like *mtr* locus. MLST ST9363 was the predominant strain type of isolates with MICs of 2–4 μg/mL identified in Australia during 2017 and the United States during 2014–2017 (*24*,*25*,*35*). We found that MLST ST9363 isolates from Argentina shared a high level of genomic similarity with the ST9363 clones reported in Australia, the United States, Canada, Norway, and Brazil, indicating that importation and dissemination has occurred. Those data further support the hypothesis that *N. gonorrhoeae* isolates carrying a mosaic-like *mtr* locus contribute to the emergence of isolates with low-level resistance to azithromycin in many countries (*24*,*25*). Isolates with MICs of >256 μg/mL have recently reemerged in Argentina (*20*). Those isolates belonged to clade 2 and were distinguished by the mosaic-like *mtr* locus and the A2059G mutation in all 4 23S rRNA gene alleles. The phylogenetic tree showed that these isolates were closely related to isolates from Norway (mean 10.2 SNPs) that also had MICs of >256 μg/mL, suggesting that strains carrying a mosaic-like *mtr* locus and 23S rRNA A2059G mutation can disseminate internationally. Previous studies have suggested that isolates carrying the A2059G mutation or mosaic *mtr* locus have enhanced fitness; elucidating the effects of both mechanisms on *N. gonorrhoeae* evolution might help predict the emergence and spread of azithromycin resistance (*39*,*46*,*47*).

Because we received a small number of isolates from some provinces, our dataset might have been limited by selection bias. In addition, we did not have access to therapy strategies and treatment success rates, which might have provided insight into the generation of resistance or the selection of azithromycin-resistant isolates. Finally, we obtained limited data regarding patients’ sexual orientation and HIV status, but found that clade 2 strains were slightly more associated with male patients, including men who have sex with men, than clade 1 strains (100.0% vs. 92.1%). In addition, 3 patients who had infections caused by clade 2 strains were HIV-positive (data not shown). Increased awareness of the transmission dynamics of azithromycin-resistant gonococcal strains within sexual networks is crucial to confirming these observations. Continuing surveillance of the prevalence and distribution of azithromycin-resistant strains in addition to genomic monitoring using individual-level epidemiologic data should provide a more complete picture of azithromycin-resistant gonococcal strains. These data will inform public health strategies to control azithromycin-resistant *N. gonorrhoeae*.

In conclusion, the recent increase in the prevalence of azithromycin-resistant *N. gonorrhoeae* isolates in Argentina was mainly the result of the introduction and expansion of 2 clones belonging to MLST ST1580 and ST9363. The integration of appropriate STI diagnosis and antimicrobial prescription into health services combined with genomic, phenotypic, and epidemiologic gonococcal surveillance data will be critical in preventing the dissemination of gonococcal clones resistant to azithromycin, ceftriaxone, or both, and preserving the current available therapeutic option for gonorrhea.

Appendix Additional information for genomic epidemiology of azithromycin-nonsusceptible *Neisseria gonorrhoeae*, Argentina, 2005–2019.

## References

[1] Rowley J, Vander Hoorn S, Korenromp E, Low N, Unemo M, Abu-Raddad LJ, et al. Chlamydia, gonorrhoea, trichomoniasis and syphilis: global prevalence and incidence estimates, 2016. Bull World Health Organ. 2019;97:548–562P. 10.2471/BLT.18.228486 31384073 PMC6653813

[2] Wi T, Lahra MM, Ndowa F, Bala M, Dillon JR, Ramon-Pardo P, et al. Antimicrobial resistance in *Neisseria gonorrhoeae*: Global surveillance and a call for international collaborative action. PLoS Med. 2017;14:e1002344. 10.1371/journal.pmed.1002344 28686231 PMC5501266

[3] Unemo M, Lahra MM, Cole M, Galarza P, Ndowa F, Martin I, et al. World Health Organization Global Gonococcal Antimicrobial Surveillance Program (WHO GASP): review of new data and evidence to inform international collaborative actions and research efforts. Sex Health. 2019;16:412–25. 10.1071/SH19023 31437420 PMC7035961

[4] Unemo M. Current and future antimicrobial treatment of gonorrhoea - the rapidly evolving *Neisseria gonorrhoeae* continues to challenge. BMC Infect Dis. 2015;15:364. 10.1186/s12879-015-1029-2 26293005 PMC4546108

[5] Unemo M, Ross J, Serwin AB, Gomberg M, Cusini M, Jensen JS. 2020 European guideline for the diagnosis and treatment of gonorrhoea in adults. Int J STD AIDS. 2020;2020:956462420949126. 10.1177/0956462420949126 33121366

[6] World Health Organization. WHO guidelines for the treatment of *Neisseria gonorrhoeae*. 2016 [cited 2020 Dec 4]. https://www.who.int/reproductivehealth/publications/rtis/gonorrhoea-treatment-guidelines/en 27512795

[7] Fifer H, Natarajan U, Jones L, Alexander S, Hughes G, Golparian D, et al. Failure of dual antimicrobial therapy in treatment of gonorrhoea. N Engl J Med. 2016;374:2504–6. 10.1056/NEJMc1512757 27332921

[8] Whiley DM, Jennison A, Pearson J, Lahra MM. Genetic characterisation of *Neisseria gonorrhoeae* resistant to both ceftriaxone and azithromycin. Lancet Infect Dis. 2018;18:717–8. 10.1016/S1473-3099(18)30340-2 29976521

[9] Eyre DW, Sanderson ND, Lord E, Regisford-Reimmer N, Chau K, Barker L, et al. Gonorrhoea treatment failure caused by a *Neisseria gonorrhoeae* strain with combined ceftriaxone and high-level azithromycin resistance, England, February 2018. Euro Surveill. 2018;23:1800323. 10.2807/1560-7917.ES.2018.23.27.1800323 29991383 PMC6152157

[10] Jennison AV, Whiley D, Lahra MM, Graham RM, Cole MJ, Hughes G, et al. Genetic relatedness of ceftriaxone-resistant and high-level azithromycin resistant *Neisseria gonorrhoeae* cases, United Kingdom and Australia, February to April 2018. Euro Surveill. 2019;24:1900118. 10.2807/1560-7917.ES.2019.24.8.1900118 30808445 PMC6446956

[11] Williamson DA, Fairley CK, Howden BP, Chen MY, Stevens K, De Petra V, et al. Trends and risk factors for antimicrobial-resistant *Neisseria gonorrhoeae*, Melbourne, Australia, 2007 to 2018. Antimicrob Agents Chemother. 2019;63:e01221–19. 10.1128/AAC.01221-19 31383663 PMC6761556

[12] US Centers for Disease Control and Prevention. Sexually transmitted disease surveillance 2017. 2018 [cited 2020 Dec 4]. https://www.cdc.gov/std/stats17/2017-STD-Surveillance-Report_CDC-clearance-9.10.18.pdf

[13] Thakur SD, Araya P, Borthagaray G, Galarza P, Hernandez AL, Payares D, et al. Resistance to ceftriaxone and azithromycin in *Neisseria gonorrhoeae* isolates from 7 countries of South America and the Caribbean: 2010–2011. Sex Transm Dis. 2017;44:157–60. 10.1097/OLQ.0000000000000587 28178114

[14] Ministerio de Salud y Desarrollo Social. Bulletin on HIV/AIDS and STIS in Argentina no. 36 [in Spanish]. 2019 [cited 2020 Dec 4]. https://bancos.salud.gob.ar/recurso/boletin-sobre-el-vih-sida-e-its-en-la-argentina-ndeg-36

[15] Clinical and Laboratory Standards Institute. 2019. Performance standards for antimicrobial susceptibility testing: twenty-ninth informational supplement (M100–S29). Wayne (PA): The Institute; 2019.

[16] Fifer H, Cole M, Hughes G, Padfield S, Smolarchuk C, Woodford N, et al. Sustained transmission of high-level azithromycin-resistant *Neisseria gonorrhoeae* in England: an observational study. Lancet Infect Dis. 2018;18:573–81. 10.1016/S1473-3099(18)30122-1 29523496

[17] Demczuk W, Martin I, Peterson S, Bharat A, Van Domselaar G, Graham M, et al. Genomic epidemiology and molecular resistance mechanisms of azithromycin-resistant *Neisseria gonorrhoeae* in Canada from 1997 to 2014. J Clin Microbiol. 2016;54:1304–13. 10.1128/JCM.03195-15 26935729 PMC4844716

[18] Palmer HM, Young H, Winter A, Dave J. Emergence and spread of azithromycin-resistant *Neisseria gonorrhoeae* in Scotland. J Antimicrob Chemother. 2008;62:490–4. 10.1093/jac/dkn235 18552343

[19] Jacobsson S, Golparian D, Cole M, Spiteri G, Martin I, Bergheim T, et al. WGS analysis and molecular resistance mechanisms of azithromycin-resistant (MIC >2 mg/L) *Neisseria gonorrhoeae* isolates in Europe from 2009 to 2014. J Antimicrob Chemother. 2016;71:3109–16. 10.1093/jac/dkw279 27432597

[20] Galarza PG, Alcalá B, Salcedo C, Canigia LF, Buscemi L, Pagano I, et al. Emergence of high level azithromycin-resistant *Neisseria gonorrhoeae* strain isolated in Argentina. Sex Transm Dis. 2009;36:787–8. 10.1097/OLQ.0b013e3181b61bb1 19734823

[21] De Silva D, Peters J, Cole K, Cole MJ, Cresswell F, Dean G, et al. Whole-genome sequencing to determine transmission of *Neisseria gonorrhoeae*: an observational study. Lancet Infect Dis. 2016;16:1295–303. 10.1016/S1473-3099(16)30157-8 27427203 PMC5086424

[22] Harris SR, Cole MJ, Spiteri G, Sánchez-Busó L, Golparian D, Jacobsson S, et al.; Euro-GASP study group. Public health surveillance of multidrug-resistant clones of *Neisseria gonorrhoeae* in Europe: a genomic survey. Lancet Infect Dis. 2018;18:758–68. 10.1016/S1473-3099(18)30225-1 29776807 PMC6010626

[23] Grad YH, Harris SR, Kirkcaldy RD, Green AG, Marks DS, Bentley SD, et al. Genomic epidemiology of gonococcal resistance to extended-spectrum cephalosporins, macrolides, and fluoroquinolones in the United States, 2000–2013. J Infect Dis. 2016;214:1579–87. 10.1093/infdis/jiw420 27638945 PMC5091375

[24] Williamson DAF, Chow EPF, Gorrie CL, Seemann T, Ingle DJ, Higgins N, et al. Bridging of *Neisseria gonorrhoeae* lineages across sexual networks in the HIV pre-exposure prophylaxis era. Nat Commun. 2019;10:3988. 10.1038/s41467-019-12053-4 31488838 PMC6728426

[25] Gernert KM, Seby S, Schmerer MW, Thomas JC IV, Pham CD, Cyr SS, et al.; Antimicrobial-Resistant Neisseria gonorrhoeae Working Group *. Azithromycin susceptibility of *Neisseria gonorrhoeae* in the USA in 2017: a genomic analysis of surveillance data. Lancet Microbe. 2020;1:e154–64. 10.1016/S2666-5247(20)30059-8 33005903 PMC7527259

[26] Gianecini RA, Zittermann S, Oviedo C, Galas M, Pardo PR, Allen VG, et al. Use of whole genome sequencing for the molecular comparison of *Neisseria gonorrhoeae* isolates with decreased susceptibility to extended spectrum cephalosporins from 2 geographically different regions in America. Sex Transm Dis. 2019;46:548–55. 10.1097/OLQ.0000000000001011 31295224

[27] World Health Organization. Laboratory diagnosis of sexually transmitted infections, including human immunodeficiency virus. 2013 [cited 2020 Dec 4]. http://apps.who.int/iris/bitstream/10665/85343/1/9789241505840_eng.pdf

[28] Gianecini RA, Golparian D, Zittermann S, Litvik A, Gonzalez S, Oviedo C, et al.; Gonococcal Antimicrobial Susceptibility Surveillance Programme-Argentina (GASSP-AR) Working Group. Genome-based epidemiology and antimicrobial resistance determinants of *Neisseria gonorrhoeae* isolates with decreased susceptibility and resistance to extended-spectrum cephalosporins in Argentina in 2011-16. J Antimicrob Chemother. 2019;74:1551–9. 10.1093/jac/dkz054 30820563

[29] Hunt M, Mather AE, Sánchez-Busó L, Page AJ, Parkhill J, Keane JA, et al. ARIBA: rapid antimicrobial resistance genotyping directly from sequencing reads. Microb Genom. 2017;3:e000131. 10.1099/mgen.0.000131 29177089 PMC5695208

[30] Martin IM, Ison CA, Aanensen DM, Fenton KA, Spratt BG. Rapid sequence-based identification of gonococcal transmission clusters in a large metropolitan area. J Infect Dis. 2004;189:1497–505. 10.1086/383047 15073688

[31] Demczuk W, Sidhu S, Unemo M, Whiley DM, Allen VG, Dillon JR, et al. *Neisseria gonorrhoeae* sequence typing for antimicrobial resistance, a novel antimicrobial resistance multilocus typing scheme for tracking global dissemination of *N. gonorrhoeae* strains. J Clin Microbiol. 2017;55:1454–68. 10.1128/JCM.00100-17 28228492 PMC5405263

[32] Nguyen LT, Schmidt HA, von Haeseler A, Minh BQ. IQ-TREE: a fast and effective stochastic algorithm for estimating maximum-likelihood phylogenies. Mol Biol Evol. 2015;32:268–74. 10.1093/molbev/msu300 25371430 PMC4271533

[33] Hadfield J, Croucher NJ, Goater RJ, Abudahab K, Aanensen DM, Harris SR. <jrn>33. Hadfield J, Croucher NJ, Goater RJ, Abudahab K, Aanensen DM, Harris SR. Phandango: an interactive viewer for bacterial population genomics. [</jrn>]. Bioinformatics. 2018;34:292–3. 10.1093/bioinformatics/btx610 29028899 PMC5860215

[34] Pommier T, Canbäck B, Lundberg P, Hagström A, Tunlid A. RAMI: a tool for identification and characterization of phylogenetic clusters in microbial communities. Bioinformatics. 2009;25:736–42. 10.1093/bioinformatics/btp051 19223450 PMC2654800

[35] Thomas JC, Seby S, Abrams AJ, Cartee J, Lucking S, Vidyaprakash E, et al.; Antimicrobial-Resistant Neisseria gonorrhoeae Working Group. Evidence of recent genomic evolution in gonococcal strains with decreased susceptibility to cephalosporins or azithromycin in the United States, 2014–2016. J Infect Dis. 2019;220:294–305. 10.1093/infdis/jiz079 30788502 PMC6581898

[36] Golparian D, Bazzo ML, Golfetto L, Gaspar PC, Schörner MA, Schwartz Benzaken A, et al.; Brazilian-GASP Network. Genomic epidemiology of *Neisseria gonorrhoeae* elucidating the gonococcal antimicrobial resistance and lineages/sublineages across Brazil, 2015-16. J Antimicrob Chemother. 2020;75:3163–72. 10.1093/jac/dkaa318 32785692

[37] Alfsnes K, Eldholm V, Olsen AO, Brynildsrud OB, Bohlin J, Steinbakk M, et al. Genomic epidemiology and population structure of *Neisseria gonorrhoeae* in Norway, 2016-2017. Microb Genom. 2020;6:e000359. 10.1099/mgen.0.000359 32213251 PMC7276708

[38] Unemo M, Shafer WM. Antimicrobial resistance in *Neisseria gonorrhoeae* in the 21st century: past, evolution, and future. Clin Microbiol Rev. 2014;27:587–613. 10.1128/CMR.00010-14 24982323 PMC4135894

[39] Wadsworth CB, Arnold BJ, Sater MRA, Grad YH. Azithromycin resistance through interspecific acquisition of an epistasis-dependent efflux pump component and transcriptional regulator in *Neisseria gonorrhoeae.* MBio. 2018;9:e01419–18. 10.1128/mBio.01419-18 30087172 PMC6083905

[40] Rouquette-Loughlin CE, Reimche JL, Balthazar JT, Dhulipala V, Gernert KM, Kersh EN, et al. Mechanistic basis for decreased antimicrobial susceptibility in a clinical isolate of *Neisseria gonorrhoeae* possessing a mosaic-like *mtr* efflux pump locus. MBio. 2018;9:e02281–18. 10.1128/mBio.02281-18 30482834 PMC6282211

[41] Ministerio de salud y ambiente de la nación. Management guide for sexually transmitted infections [in Spanish]. Buenos Aires: El Ministerio; 2004.

[42] Sociedad Argentina de Infectologí a. First consensus on the diagnosis, treatment, and prevention of sexually transmitted infections [in Spanish]. 2011 [cited 2020 Dec 4]. https://drive.google.com/file/d/1VqW7USdEyO5FkJXwB239f8pudQ9MbDUr/view

[43] Kong FYS, Horner P, Unemo M, Hocking JS. Pharmacokinetic considerations regarding the treatment of bacterial sexually transmitted infections with azithromycin: a review. J Antimicrob Chemother. 2019;74:1157–66. 10.1093/jac/dky548 30649333

[44] St Cyr S, Barbee L, Workowski KA, Bachmann LH, Pham C, Schlanger K, et al. Update to CDC’s treatment guidelines for gonococcal infection, 2020. MMWR Morb Mortal Wkly Rep. 2020;69:1911–6. 10.15585/mmwr.mm6950a6 33332296 PMC7745960

[45] Fifer H, Saunders J, Soni S, Sadiq ST, FitzGerald M. 2018 UK national guideline for the management of infection with *Neisseria gonorrhoeae.* Int J STD AIDS. 2020;31:4–15. 10.1177/0956462419886775 31870237

[46] Handing JW, Ragland SA, Bharathan UV, Criss AK. The MtrCDE efflux pump contributes to survival of *Neisseria gonorrhoeae* from human neutrophils and their antimicrobial components. Front Microbiol. 2018;9:2688. 10.3389/fmicb.2018.02688 30515136 PMC6256084

[47] Zhang J, van der Veen S. *Neisseria gonorrhoeae* 23S rRNA A2059G mutation is the only determinant necessary for high-level azithromycin resistance and improves in vivo biological fitness. J Antimicrob Chemother. 2019;74:407–15. 10.1093/jac/dky438 30376120

