# Supplementary material for: Genomic Epidemiology of Azithromycin-Nonsusceptible Neisseria gonorrhoeae, Argentina, 2005–2019
Source: Emerg Infect Dis. 2021 Sep;27(9):2369–78. doi: 10.3201/eid2709.204843 (PMC8386799; doi:10.3201/eid2709.204843)
Supplement: Appendix — Additional information for genomic epidemiology of azithromycin-nonsusceptible Neisseria gonorrhoeae, Argentina, 2005–2019. [file 20-4843-Techapp-s1.pdf]

Article DOI: https://doi.org/10.3201/eid2709.204843

Genomic Epidemiology of Azithromycin-Nonsusceptible *Neisseria gonorrhoeae*, Argentina, 2005–2019

Appendix

Appendix Methods

Antimicrobial Susceptibility Testing

We subcultured N. gonorrhoeae isolates on Difco GC medium agar base (Becton, Dickinson and Company, https://www.bd.com) supplemented with 1% Britalex enrichment supplement (Laboratorios Britania S.A., https://www.britanialab.com) for 18–24 h at 35°C in a humid 5% carbon dioxide–enriched atmosphere before conducting antimicrobial susceptibility testing. We determined the MICs of azithromycin, ceftriaxone, cefixime, ciprofloxacin, spectinomycin, benzylpenicillin, tetracycline, and gentamicin (MilliporeSigma, https://www.sigmaaldrich.com) using the agar dilution method (Reference *1* in Appendix). We interpreted the MICs using CLSI breakpoints (*15*), except for gentamicin, for which we used previously published interpretive criteria (Reference *2* in Appendix). We used the N. gonorrhoeae strain ATCC 49226 and 8 WHO reference strains documented in 2008 for quality control (*15*, Reference *3* in Appendix).

Whole-Genome Sequencing

We extracted genomic DNA using QIAamp DNA Mini Kit (QIAGEN, https://www.qiagen.com) according to the manufacturer's instructions. We determined DNA concentration using the Qubit 2.0 Fluorometer (Thermo Fisher Scientific, https://www.thermofisher.com) and stored samples at −20°C. We conducted WGS on all isolates using the Nextera XT DNA library preparation kit and the MiSeq Platform (Illumina, https://www.illumina.com) according to the manufacturer's instructions. We assessed the quality of the sequences using FastQC version 0.11.9 (Babraham Institute,

http://www.bioinformatics.babraham.ac.uk) and identified contaminants using Kraken 2 version 2.08 (Johns Hopkins University, http://ccb.jhu.edu). We assembled de novo reads using Unicycler version 0.4.8, which is based on SPAdes version 3.13.0 (Reference *4* in Appendix), and assessed assembly quality using Quast version 5.0.2 (Reference *5* in Appendix). On average, the numbers of contigs was 103 and the N50 contig length (i.e., the length for which half of the bases of a draft genome are situated in contigs of that length or longer) was 48,458 bp.

Additional References

1. Clinical and Laboratory Standards Institute. Methods for dilution antimicrobial susceptibility tests for bacteria that grow aerobically: approved standard-eleventh edition. Wayne (PA): The Institute; 2019.

2. Gianecini R, Oviedo C, Irazu L, Rodríguez M, Galarza P; GASSP-AR Working Group. Comparison of disk diffusion and agar dilution methods for gentamicin susceptibility testing of *Neisseria gonorrhoeae*. Diagn Microbiol Infect Dis. 2018;91:299–304. PubMed https://doi.org/10.1016/j.diagmicrobio.2018.03.005

3. Unemo M, Fasth O, Fredlund H, Limnios A, Tapsall J. Phenotypic and genetic characterization of the 2008 WHO *Neisseria gonorrhoeae* reference strain panel intended for global quality assurance and quality control of gonococcal antimicrobial resistance surveillance for public health purposes. J Antimicrob Chemother. 2009;63:1142–51. PubMed https://doi.org/10.1093/jac/dkp098

4. Antipov D, Korobeynikov A, McLean JS, Pevzner PA. hybridSPAdes: an algorithm for hybrid assembly of short and long reads. Bioinformatics. 2016;32:1009–15. PubMed https://doi.org/10.1093/bioinformatics/btv688

<jrn>5. Gurevich A, Saveliev V, Vyahhi N, Tesler G. QUAST: quality assessment tool for genome assemblies. Bioinformatics. 2013;29:1072–5.<jrn>

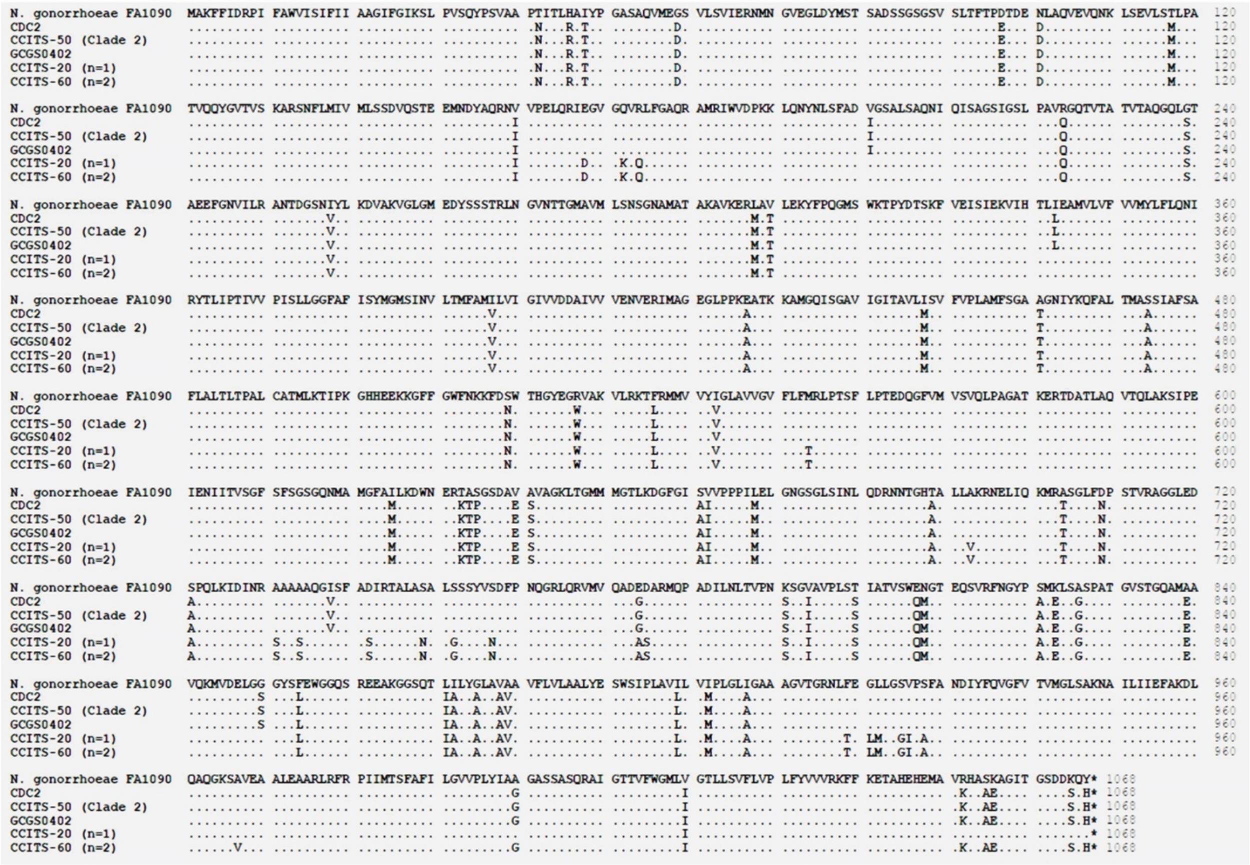

Appendix Figure. Amino acid sequences of mosaic MtrD loci in *N. gonorrhoeae* isolates, Argentina, 2005–2019. Amino acid sequences are aligned to the wildtype sequence of *N. gonorrhoeae* FA1090 (GenBank accession no. AE004969) and previously described mosaic MtrD sequences from CDC-2 and GCGS0834 strains (*44*,*45*). Dashes indicate amino acid residues identical to those of FA1090.
